# Feedback-Related ERP Components Are Modulated by Social Distance during Non-Contingent Evaluation of Someone Else’s Performance

**DOI:** 10.1371/journal.pone.0156656

**Published:** 2016-05-27

**Authors:** Erwin Rogelio Villuendas-González, Andrés Antonio González-Garrido

**Affiliations:** 1 Facultad de Psicología, Universidad Michoacana de San Nicolás de Hidalgo, Morelia, México; 2 Laboratorio de Neurofisiología Clínica, Instituto de Neurociencias, Centro Universitario de Ciencias Biológicas y Agropecuarias, Universidad de Guadalajara, Guadalajara, México; 3 Hospital Civil Fray Antonio Alcalde, Universidad de Guadalajara, Guadalajara, México; University of Tuebingen Medical School, GERMANY

## Abstract

Performance monitoring depends on cortical structures that are also activated in vicarious monitoring. While many experiments have shown that vicarious and on-line monitoring have a similar basis, most such experiments have focused on simple tasks. In order to assess the effect of non-contingent feedback on vicarious monitoring, 23 young volunteer adults were evaluated: in one session, they performed a rule-based category formation task, receiving no feedback on their performance. In a second session, Event Related Potentials (ERPs) were obtained while participants passively reviewed performances attributed to themselves and peers they had previously rated as either socially close or distant. Feedback Related Negativity (FRN) and Feedback Related P300 (fP300) components were analyzed with respect to feedback valence and agent. Results show that both components can be elicited through non-contingent feedback related to prior performance. In addition, FRN waves are modulated by the valence of the feedback, and fP300 is modulated by the agent to whom performance feedback is attributed. This experiment constitutes a novel approach to the evaluation of ERP correlates of vicarious monitoring through non-contingent feedback and its relations to empathy processing.

## Introduction

Every action we perform depends upon a complex system in which both efferent (motor) and afferent (sensitive) systems participate establishing feedback loops. In order to be more precise, our performance relies on systems that involve feedback loops at different stages: from a superior level in which motor programming is regulated before execution through action planning, to feedback that occurs in an interpersonal context, as when someone else offers information on the quality of our execution.

Performance monitoring studies have provided important data on the underlying anatomical bases and physiological processes involved in this phenomenon. A complex network has consistently been shown to underlie performance monitoring: rapid responses depend on the right dorsolateral prefrontal cortex (DLPFC) and anterior cingulate cortex (ACC), error and conflict processing rely on medial structures (such as the ACC and the supplementary motor cortex), and task maintenance depends upon the DLPFC [1].

Brain structures related to feedback processing have also been widely studied: positive feedback has been shown to activate the ventral striatum (nucleus accumbens) while negative feedback activates the rostral portion of the motor cingulate area, the anteroinferior portion of the insula and the epithalamus [2]. It has been proposed the ACC plays several roles in performance monitoring: conflict signaling in information processing [3,4], error detection [5,6], and reinforcement processing [7].

This network underlying performance monitoring seems to be partially shared in vicarious (observational) monitoring; Shane, Stevens, Harenski and Kiehl [8] identified brain regions activated while observing someone else’s errors–particularly the rostral/ventral portion of the ACC and the inferior parietal cortex (IPC). These authors propose that the IPC allows the subject to distinguish between her/his own actions and those of others, while the ACC participates in the context-mediated comprehension of errors made by others. Yu and Zhou [9] obtained similar activations related to one’s own performance and that of others, further supporting the idea that similar mechanisms allow self and vicarious performance monitoring. Apps and colleagues [10] recently found that neurons in the ACC signal prediction errors when learning from the outcomes of one’s own action but also when outcomes are received from others.

In the same vein, Uddin, Iacoboni, Lange and Keenan [11] propose that the frontoparietal mirror neuron system constitutes the base that conveys information on oneself and others through motor stimulation mechanisms, while midline structures process information about others and oneself though in a more abstract and evaluative manner. The medial prefrontal cortex (mPFC) appears to mediate metacognitive processing that might be used for direct and reflected self-evaluations, depending on the demands of a specific task [12].

Event-Related Potentials (ERPs) studies, though lacking a significant spatial resolution, allow us to determine at what precise moment distinct brain regions are involved in a particular task. Psychologically, ERPs represent a neuronal manifestation of specific information processing activities associated with either a stimulus or a response [13]. Analysis of ERPs with respect to errors in performance has unveiled neural processes specifically associated with monitoring and behavioral adjustment [14]. Four ERP components have been linked to error monitoring: Error-Related Negativity (ERN or Ne), Error-Related Positivity (Pe), Feedback-Related Negativity (FRN), and Feedback related P300 (fP300).

One of the most frequently studied ERP components is error-/feedback-related negativity (FRN), which peaks at fronto-central scalp sites [15, 16, 17]. This component was first described by Miltner, Braun and Coles [18] and differentiates negative from positive feedback, thus reflecting a complex interaction between past- and present-related actions, information, thoughts and emotions [19].

Empirical evidence from studies using functional magnetic resonance imaging (fMRI), or the combination of electroencephalography (EEG) with fMRI, have suggested that FRN is generated in the posterior medial frontal cortex (pMFC) and the anterior cingulate cortex [20, 21, 22, 23, 24].

The earliest studies of FRN [18] used temporal estimation tasks and found that feedback was followed by an electrophysiological response characterized by a midline negativity that began about 250ms after feedback onset. FRN has been elicited by both current performance errors and feedback unrelated to ongoing performance, but related to recent activities [25]. Further studies, such as one by Gehring and Willoughby [26] showed that even if FRN is larger for losses compared to gains (in a gambling task), its amplitude does not necessarily depend on the magnitudes of the losses or gains. It has also been shown that FRN appears as long as feedback provides new relevant information on performance, linking it with the information from internal monitoring processes [27]. Although most FRN studies have used contingent feedback–*i*.*e*., dependent on ongoing performance–some have shown that the FRN might be obtained even if the subject is not producing a response or actively making a choice [25].

Koban, Pourtois, Bediou and Vuilleumier [28] evaluated the ERP responses for feedback generated by oneself and others’ performances in cooperative and competitive contexts. They found that FRN was present for both performers and observers (although with smaller amplitudes when they were competing). These results support the notion that the subject’s own performance feedback could vary as a function of social context. If so, it could be useful to further evaluate social distance and its possible contribution to the performance monitoring.

P300, meanwhile, is a stimulus-locked ERP component that peaks at around 300–600 ms after stimulus presentation on posterior/parietal scalp sites. P300 seems to reflect attentional reallocation processes, and was originally associated with “context updating of a stimulus representation” [29, 30]; *i*.*e*., an updating of the internal environmental representation [31]. According to the model proposed by Polich [32], P300 includes an early attentional process that originates in a representational change in frontal working memory and produces the P3a component, leading to the signal being transmitted to temporal and parietal structures associated with the P3b component. A wide variety of stimuli have been used to elicit the P300 component, among them performance feedback, in which case the wave has been described as a feedback-related P300 (or fP300). In an experiment that compared feedback related to one’s own performance to that associated with someone else’s performance, fP300 amplitude varied depending on the valence of the feedback and the interpersonal relationship between the observer and the person that performed the task [33, 34]. A study by Gray, Ambady, Lowenthal and Deldin [35] showed that information related to one’s own performance or that of or socially-close persons produced greater P300 waves, while Bäuchl [36] found that the amplitude of fP300 in association with monetary losses varied depending on the person to whom the losses were attributed, and that the amplitude also correlated positively with scores on an empathy scale.

The aim of the present study, then, was to explore the characteristics of two feedback-related components (FRN and fP300), when elicited through performance errors attributed to the participant or to peers that the participant had previously judged as being socially-close or socially-distant. Our predictions were that both components related to feedback processing would be obtained through non-contingent feedback and that they would be modulated both by the valence of the feedback (as literature has widely shown for contingent feedback) and by the social distance between the participant and the peers whose performances were being reviewed (as the allocation of attentional resources is likely to be related to the agent whose performance one is reviewing).

## Materials and Methods

### Participants

An open invitation was made to a group of healthy undergraduate students in which information about the experiment was kept to a minimum. All subjects participated voluntarily in the study and were informed that there would be a payment of an amount that would depend on their performance. 41 subjects attended to the first session (completing task 1 and questionnaires). Only 26 were asked to attend to the second session (completing task 2 during EEG recording), as the rest of participants did not label enough of their peers as either close or distant, (a condition in order to fulfill task 2, as is explained in the Procedure section). Three subjects were not included in the analysis as during outbriefing they manifested doubts regarding the veracity of trials attributed to their peers. A repeated measures experimental design was used with the remaining 23 participants (14 women, average age = 19.5, SD = 0.67). They were all attending the first year of the psychology undergraduate program at a public university and they were all paid the equivalent of $15 USD for attending the two experimental sessions. The nature of the experiment was disclosed only at the conclusion of the second session and participants were encouraged not to share any of the information with their peers until the conclusion of the study.

### Experimental tasks

#### Task 1

**Fig 1:**
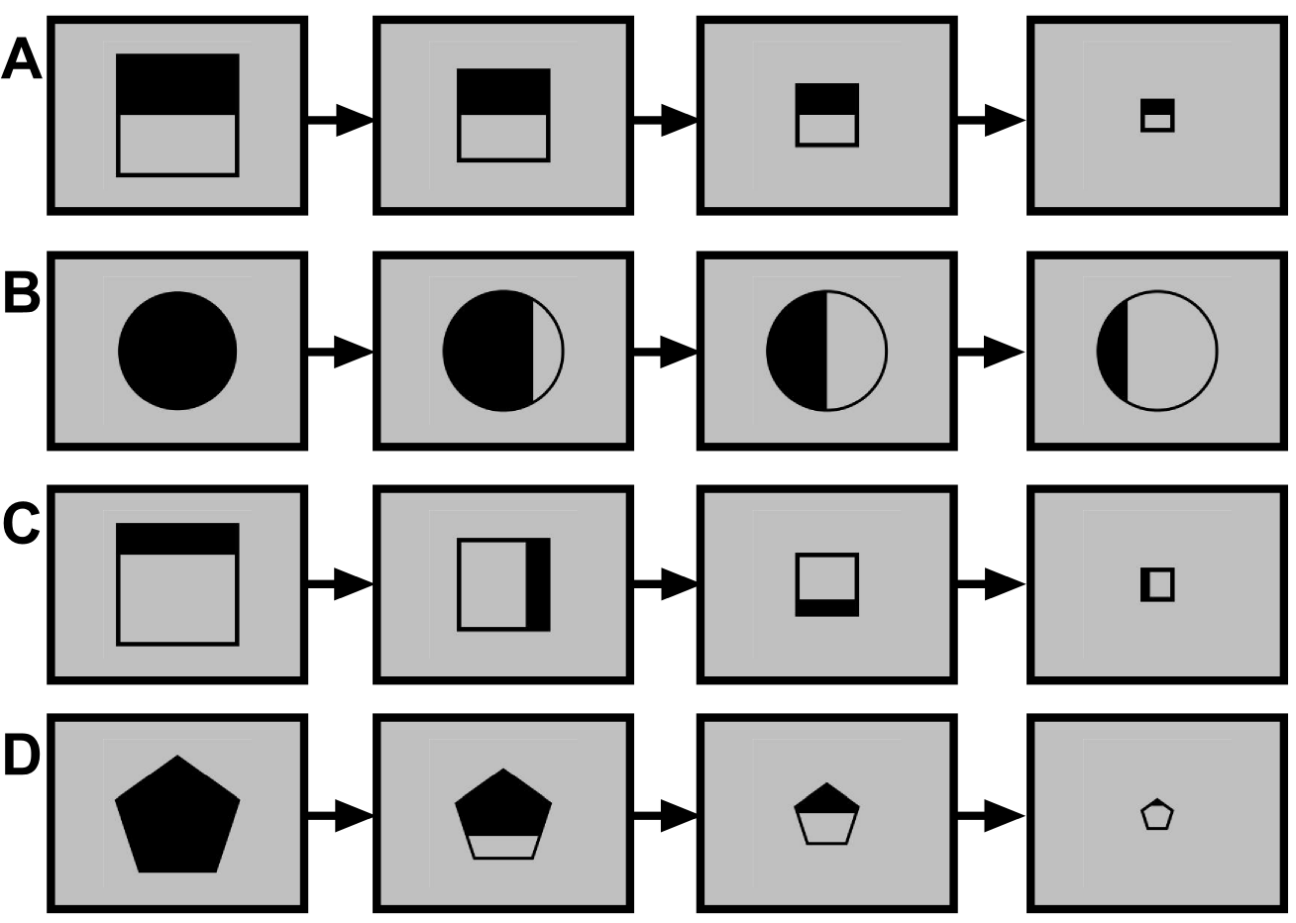
Samples of different series. A and B show series with variation of one attribute (decreasing size in A, decreasing fill in B) while C and D show series with variation of two attributes (decreasing size and fill rotating in C, decreasing size and fill decrease in D).

This task had 6 blocks of 30 trials each. Every trial was made up of a series of 4 images shown sequentially. Stimuli appeared on a computer screen at a distance of 60 cm from the subject. Simple black colored over a gray background geometric figures (with similar visual dimensions averaging 4x4 cm) were used (arrows, squares, circles, rectangles and pentagons). Participants were instructed to indicate, by pressing a key as quickly as possible, whether the last image shown satisfied, or failed to satisfy, the criterion established by the three earlier images (see top of Fig 1). In half of the trials, only one variable defined the series (e.g. squares increasing in size; arrows turning clockwise); while in the other half, two variables were manipulated simultaneously (e.g. half-filled squares decreasing in size while the filling turned clockwise). Fig 1 shows samples of both kinds of series. The presentation order of the trials was randomized. Apart from this main instruction, participants received no other hints on how to respond to the task and no feedback related to their performance.

#### Task 2

This task had a total of 225 trials divided in 6 blocks–presented in 3 pairs separated by rest periods–and was administered with simultaneous EEG recording. This amount of trials resulted in 25 trials per condition (3 valence conditions x 3 agent conditions).

Participants were instructed to passively review performance on Task 1. Prior to beginning the revision process of each pair of blocks, a screen informed the name of the person whose trials would be reviewed. The names could pertain to: a) the participant her/himself; b) 2 classmates that she/he had previously ranked with low scores on the social distance questionnaire (*i*.*e*., socially-close); and, c) 2 classmates ranked with high scores on the same social distance questionnaire (*i*.*e*., socially-distant). Trials for the “own performance” blocks were randomly chosen from the participant’s real performance on Task 1, attempting to balance simple and complex trials. Trials for both “close peer” and “distant peer” blocks were fabricated using balanced simple and complex trials from Task 1.

**Fig 2:**
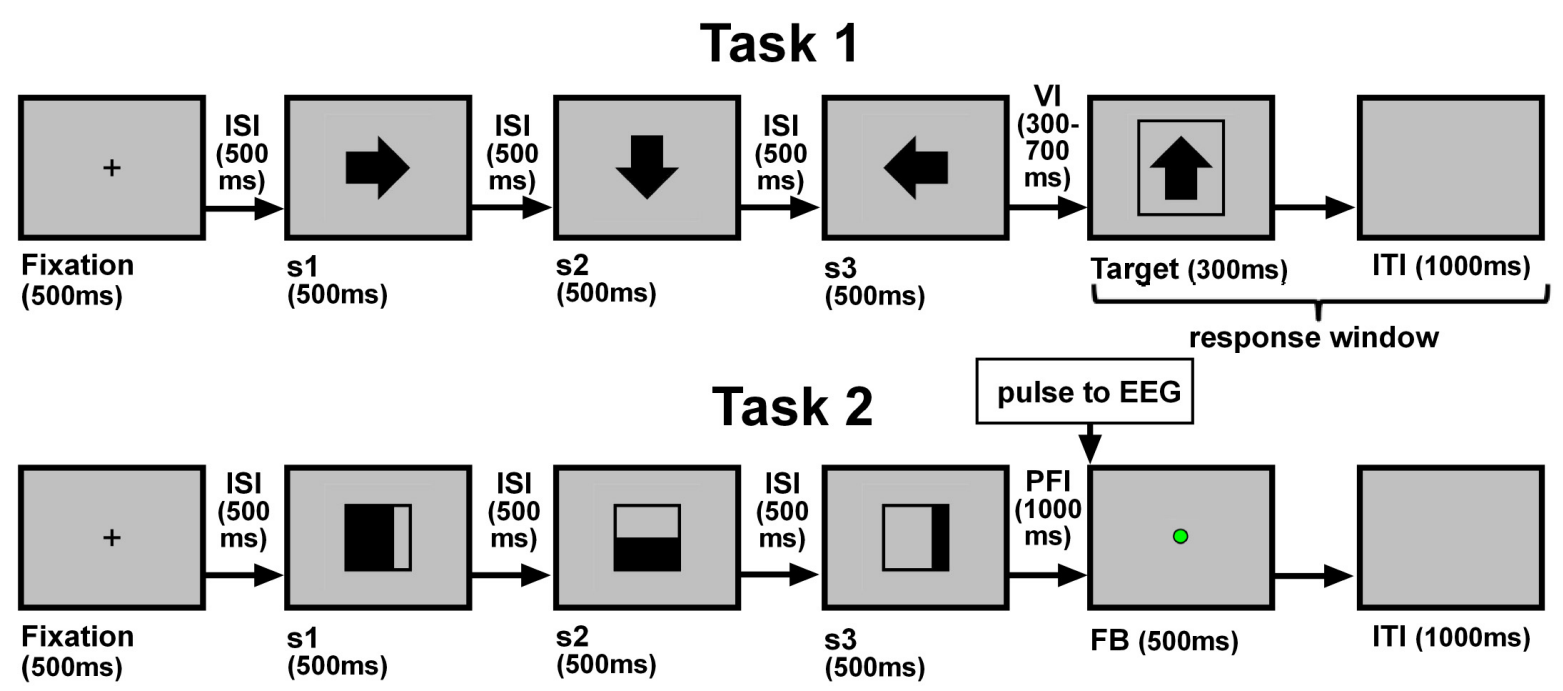
Structure of Tasks 1 and 2. In Task 1 (top): Images s1 through s3 represent arrows turning clockwise. Subject has to decide if the “Target” image follows the principle established by the previous three images. In Task 2 (bottom): Images s1 through s3 represent a sequence to which subjects has responded as part of Task 1. Feedback (FB) is given by a colored circle that codes the adequacy of the response given in that particular trial. (ISI, Inter-stimulus Interval; VI, Variable Interval; PFI, Pre-feedback Interval; ITI, Inter-trial interval).

The order of presentation of the blocks included a subject-offset to counterbalance the conditions. As in this task participants did not have to respond, a simple two-choice gambling task block was used between blocks in order to maintain attention and arousal levels during the session. Each trial had a similar structure to those of Task 1, but the last image was replaced with a colored isoluminant dot that acted as feedback on performance by indicating an error (red), a correct response (green), or non-informative feedback (neutral; gray, see bottom of Fig 2). The pulses of ERP window selection were locked to the presentation of this feedback.

### Procedure

In the first experimental session, the subjects filled out a questionnaire that measured social distance within the group. In this questionnaire they were asked to estimate how close they felt to each member of their group using a 7-point scale (from socially-close to socially-distant). The procedure followed was similar to those used in the *Social Networks Inventory* [37], but in the present case, instead of asking the participant to list the people that are part of her/his social network, we provided them with a list of their peers that had been part of the same group for at least the past year. As a result, lists of socially-close (scores of 1 or 2) and socially-distant (6 or 7) classmates were obtained for each participant.

Participants performed Task 1 while seated in a sound- and light-attenuated room. All subjects were adequately trained before performing the task. Stimulus delivery, response collection and data acquisition onset were all synchronized and controlled by the E-prime 2.0 software (Psychology Software Tools). Behavioral responses were registered through a Psychology Software Tools Serial Response Box.

Two weeks after the first session, a second session took place in order to perform Task 2, which included simultaneous EEG recording. Subjects also completed Task 2 while seated in a dimly-lit, sound-attenuated room. The task lasted about 30 minutes. Upon completion, subjects were given a brief explanation of the nature of the experiment. Each participant reviewed a total of six blocks of 30 trials each. Blocks were attributed to their own performance (2), performance from peers they had ranked as socially-close (2), and peers ranked as socially-distant (2) (blocks attributed to themselves contained their actual performance, while those attributed to their peers contained fabricated performances, to control for number of correct and erroneous responses). Whenever a participant mentioned during outbriefing being suspicious about the authenticity of the observed performance, the data involved was excluded from further analyses. Participants were encouraged not to share any of the information about the contents of the session with their peers.

### EEG recording

Electroencephalographic activity was recorded from the Fp1, Fp2, F7, F3, Fz, F4, F8, T3, C3, Cz, C4, T4, T5, P3, Pz, P4, T6, O1 and O2 scalp sites through an elastic cap (Electro-cap International, Inc.). Additional 10-mm diameter gold disk electrodes (Grass Type E5GH, Astro-Med, Inc.) were placed to record electrooculograms from the infraocular ridge of the left eye and the outer canthus of the right eye, while using linked earlobes as reference. Inter-electrode impedances were maintained below 5kΩ, with a sampling rate of 200 Hz. Signals were amplified at a bandpass filter of 0.5-30Hz (3-dB cutoff point of 6-dB/octave roll-off curves) in a MEDICID-04 system. Finally, data were filtered off-line with a 0.5-20Hz digital filter.

Single trial data were stored off-line for averaging and analysis. Individual and group grand averages were built only for 1.200-s EEG time epochs. Epochs with artifacts were rejected by visual inspection. Twenty artifact-free EEG epochs of 1200 ms (200 ms as pre-stimulus baseline) were obtained from each condition and participant. Data files are electronically available [38].

### Data analysis

**Fig 3:**
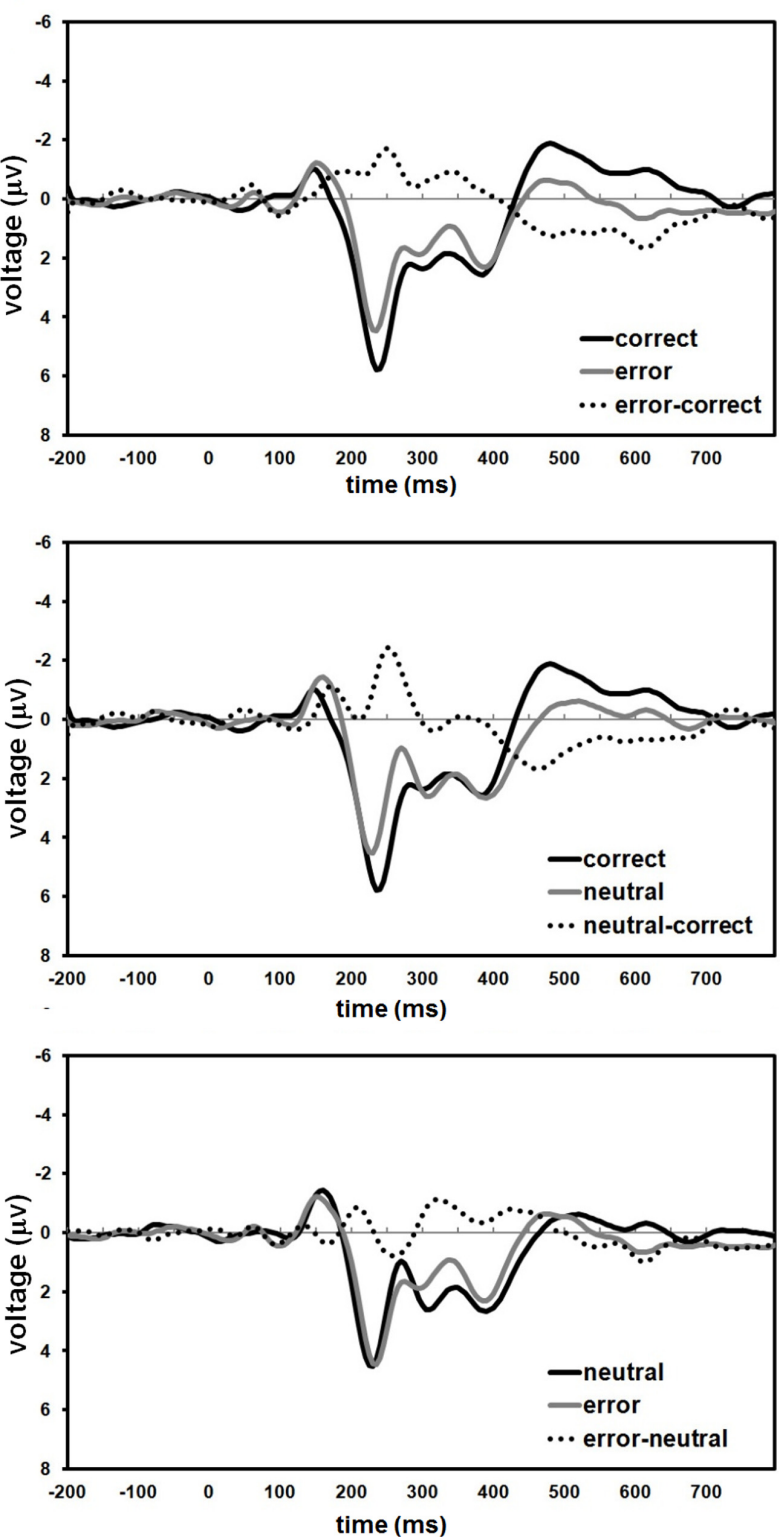
Difference ERP waveforms at Fz. (Top) error minus correct wave; (Middle) neutral minus correct wave; (Bottom) error minus neutral wave. The FRN can be observed in the two first subtractions (top and middle) but not on the third one (bottom), as neutral and error waveforms are similar in the 200-300ms window. Agent conditions are collapsed throughout the plots to show differences between valences.

**Fig 4:**
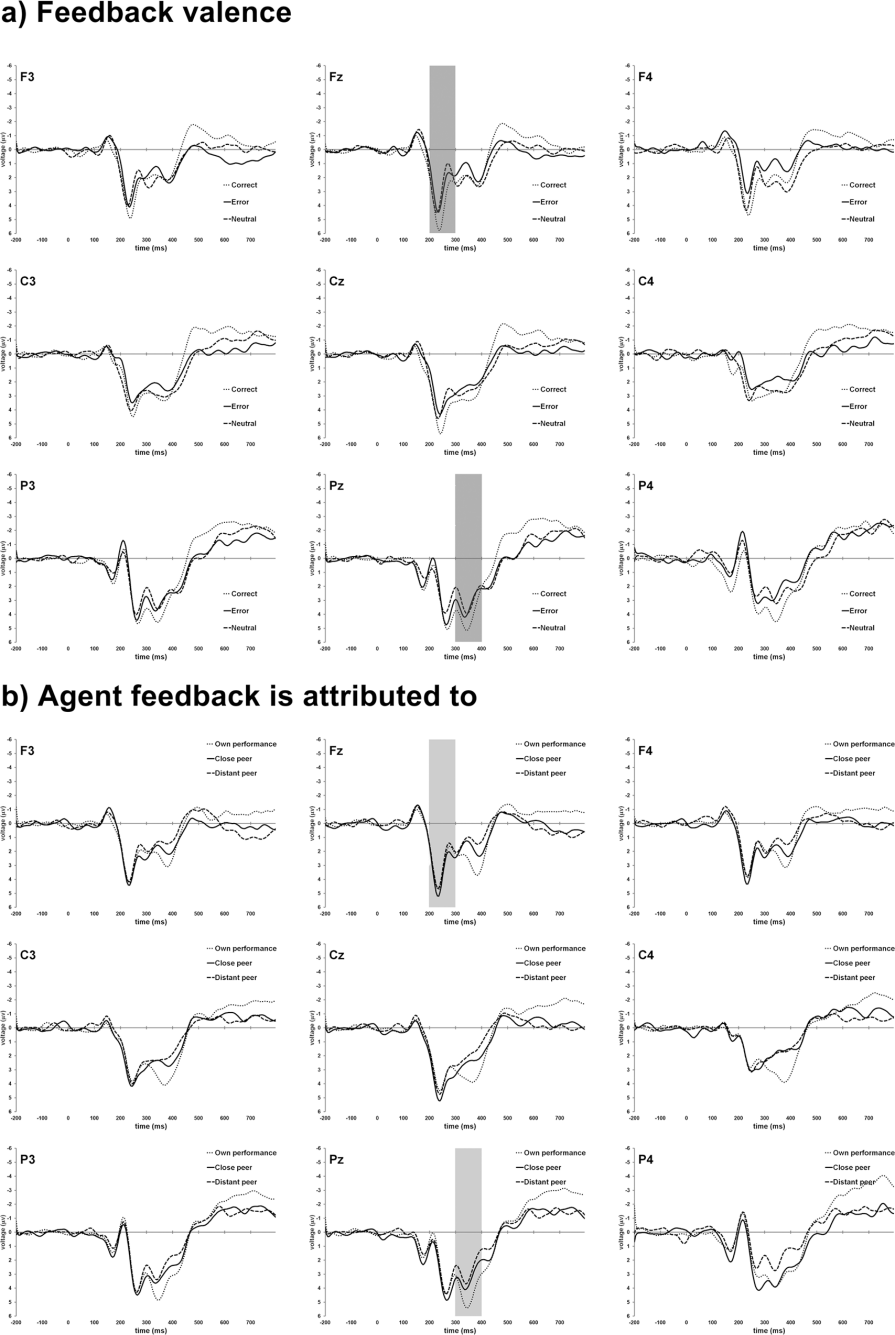
Grand-averaged ERP waveforms. F3, Fz, F4, C3, Cz, C4, P3, Pz and P4 sites are depicted representing the different feedback conditions. Top figures (a) depict the three different valences (different agents are collapsed) while bottom figures (b) depict different agents (different valences are collapsed). The 200–300 ms window at Fz and the 300–400 ms window at Pz used to respectively obtain the FRN and fP300 are highlighted.

Upon visual inspection of difference waves obtained by subtracting error-feedback from correct feedback, FRN was identified as a peak in the difference wave at 250ms after feedback, and was most prominent at Fz (Fig 3). Therefore, FRN was quantified as the average voltage in the 200-300ms window, at Fz. Grand average plots showed a P300 wave peaking 350ms after feedback, so feedback-related P300 was quantified as the average voltage within the 300-400ms window, at Pz. Data from all voltage variables were checked for normality using the Shapiro-Wilk test, resulting in W values ranging from .914 to .982 (*df* = 23), all with *p*>.05. For both components, separate repeated measures ANOVAs using two factors for condition (3 valences x 3 agents) were performed. Greenhouse-Geisser corrections were used as needed and Bonferroni corrections were applied for multiple comparisons. Scalp distribution of grand averages is shown in Fig 4.

## Results

### Task 1

Response time average was 652ms (SD = 125ms) for simple trials and 724ms (SD = 124ms) for complex trials. Average accuracy was .79 (SD = .11) for simple trials, and .50 (SD = .08) for complex trials. This performance resulted in an average of 80 correct trials and 40 error trials (of which 25 correct and 25 error trials were used in the performance review in Task 2).

### Task 2

#### Feedback-related negativity (FRN)

**Fig 5:**
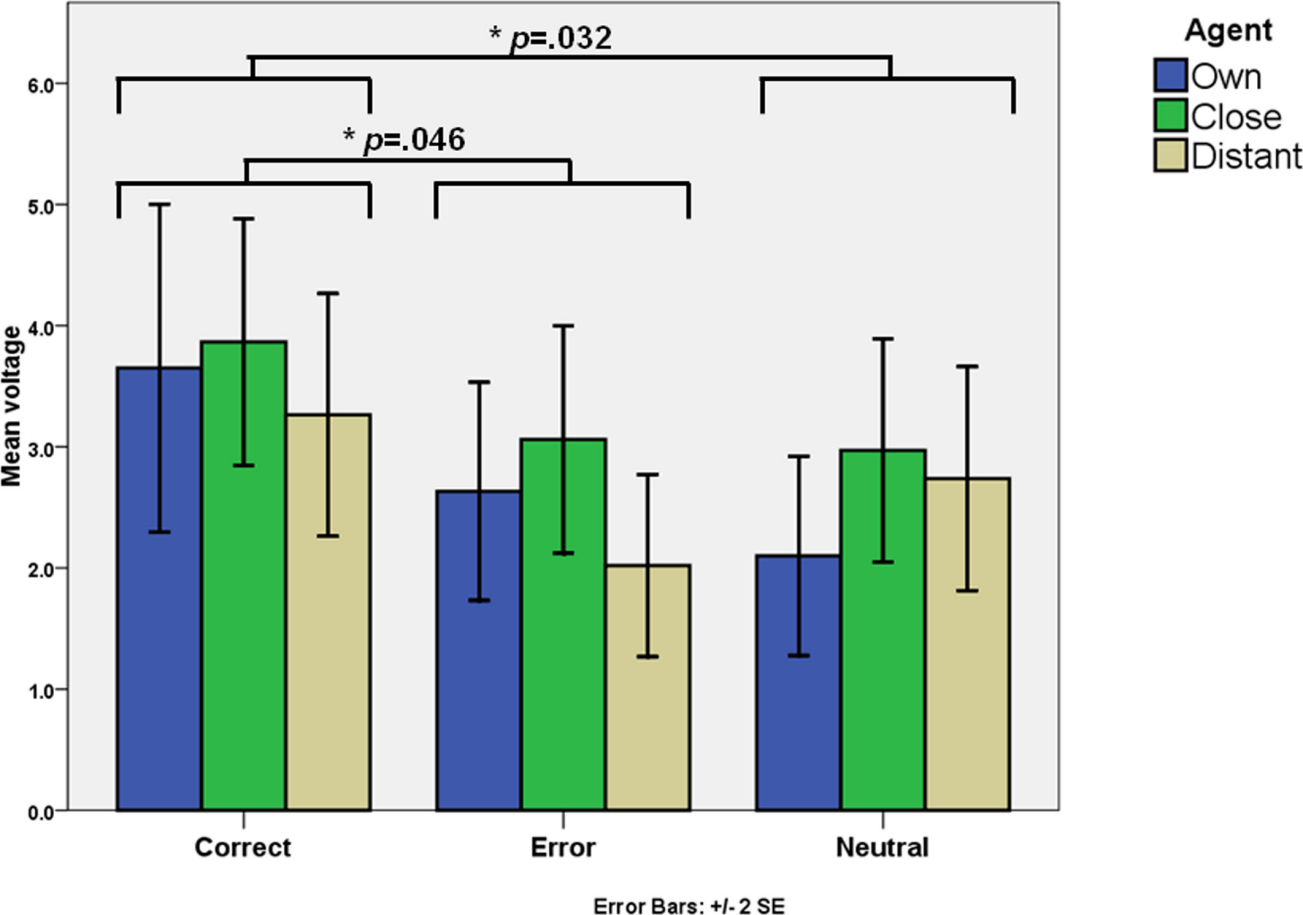
Voltages for FRN at Fz. Statistically significative differences are highlighted.

A 3 (Valence Correct vs. Error vs. Neutral) x 3 (Agent Own vs. Close vs. Distant) ANOVA showed no agent effect for this component (*F*(2,44) = 2.40, *p* = .10), but a valence effect was identified (*F*(2,44) = 5.55, *p =* .007, *η*^2^_*p*_ = .20). Lower voltages were observed for error feedback (M = 2.57μv, SD = 2.96) and neutral feedback (M = 2.60μv, SD = 2.02), compared to correct feedback (M = 3.60μv, SD = 3.96). Pairwise comparisons showed significant differences for correct *vs*. error (*p* = .046), and correct *vs*. neutral (*p* = .032). Error and neutral feedback had similar voltages, as shown in Fig 5.

#### Feedback related P300 (fP300)

**Fig 6:**
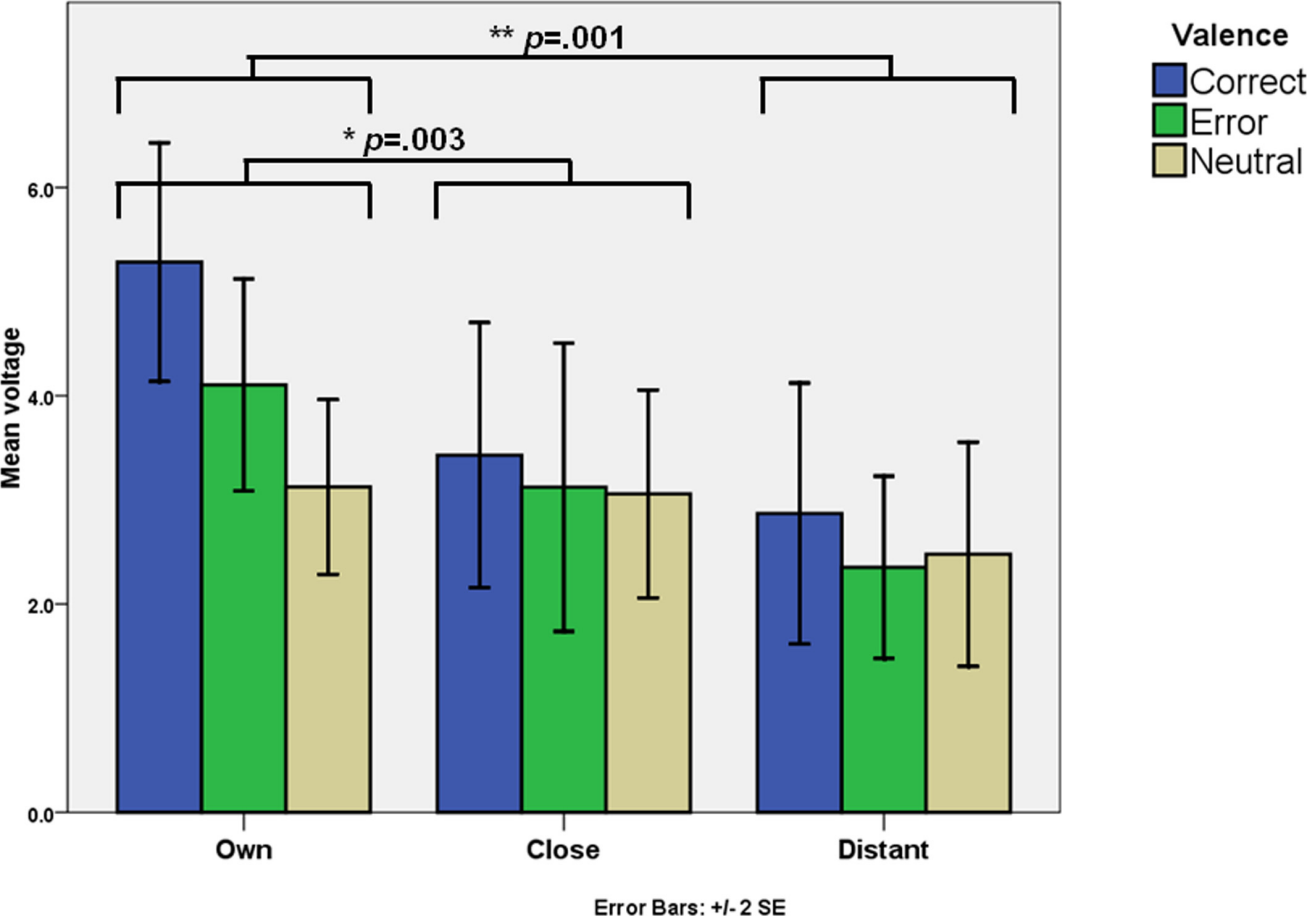
Voltages for fP300 at Pz. Statistically significative differences are highlighted.

In a 3 (Valence Correct vs. Error vs. Neutral) x 3 (Agent Own vs. Close vs. Distant) ANOVA, an agent effect was found (*F*(1.57,34.59) = 12.78, p < .001, *η*^2^_*p*_ = 0.37. Higher voltages were observed when subjects reviewed their own performance (M = 4.17μv, SD = 3.24) compared to that of a close peer (M = 3.20μv, SD = 3.69), and that of a distant peer (M = 2.57μv, SD = 4.89). Pairwise comparisons showed that only reviewing their own performance differed from reviewing someone else’s performance: (own *vs*. close, *p* = .003; own *vs*. distant, *p* < .001) and that reviewing someone else’s performance was the same regardless of the agent (close *vs*. distant, *p* = .36) (Fig 6). A small valence effect was observed as well (*F*(2,44) = 3.48, *p* = .04, *η*^2^_*p*_ = .14). The highest voltages appeared for positive feedback (M = 3.86μv, SD = 2.59), followed by negative feedback (M = 3.19μv, SD = 2.22), and then neutral feedback (M = 2.89μv, SD = 1.56).

An agent-valence interaction was identified. Simple effect analysis showed that the valence effect on voltage is due to differences in voltages for the correct and error feedback conditions: for correct feedback voltages on agents differ: own (M = 5.28μv, SD = 4.76), close (M = 3.43μv, SD = 5.28), then distant (M = 2.87μv, SD = 5.21). Pairwise comparisons show significant differences when comparing own *vs*. close (*p* = .003), and own *vs*. distant (p < .001), but not close *vs*. distant (*p* = .864). For error feedback, voltages on agents differ: own (M = 4.11μv, SD = 4.24), close (M = 3.12μv, SD = 5.76), then distant (M = 2.35μv, SD = 3.64). Pairwise comparisons showed that differences arose only in the own *vs*. distant conditions (*p* < .001). For neutral feedback, no differences between agents were found.

## Discussion

The aim of the present experiment was to study the characteristics of Feedback-Related Negativity (FRN) and Feedback-Related P300 (fP300) on a task in which performances of participants and classmates were reviewed. For this purpose, an experimental task was purposely developed to keep all trials substantially different, so that it would make sense to review performance in a later session. Our results show that on this particular task, both an FRN and an fP300 can be obtained in a non-contingent manner (not during performance, but relating to prior performance) when receiving feedback attributed both to the participant’s own performance and that of her/his peers. This allowed us to obtain insight into the effect that two variables–in this case agent (the person on whose performance the participant receives feedback), and the valence of the feedback–exercise on the ERP waveforms.

Valence of the feedback had an effect on FRN voltage that made negative and neutral feedback produce more negative voltages compared to positive feedback. These results are similar to those obtained in previous experiments [39, 40] in which neutral and negative feedback elicited similar FRNs. These authors suggest that the system that produces FRN might classify outcomes dichotomically by distinguishing whether or not the goal was met: a positive feedback elicits smaller responses (as the current goal is met) than negative feedback (goal not met) and neutral feedback would be treated the same (goal not met). In some experiments neutral feedback might elicit even larger FRN responses than an actual error feedback [40] though most of the studies report comparable amplitudes.

FRN was not modulated by agent, since similar voltages were obtained regardless of the agent performance was attributed to. In this sense, our results differ from those obtained by Carp, Halenar, Quandt, Sklar and Compton [41], who observed a modulation of FRN upon comparing the observation of friends vs. strangers during task performance. However, the present experiment differs in at least two aspects: participants reviewed performances attributed to known peers (judged as close or distant) instead of strangers, and more important, as feedback was provided in a subsequent session therefore losing its regulatory value, the difference between FRN amplitude regarding agent that has been described before [28, 33] can be interpreted as enhanced by the regulatory value of feedback: I can modify my successive responses after negative feedback (therefore having larger FRNs for my own performance) but the possibility of negative feedback to modify my behavior once the task has been completed is similar as the possibility to modify someone else’s performance, i.e. inexistent (therefore having similar FRNs for my errors and someone else’s errors). As Koban et al. [28] have noted, several authors have posited that feedback related to one’s own performance would be of greater importance because of its subsequent behavioral adjustment properties.

It would have been desirable to assess the effect of task difficulty on feedback processing by performing a separate analysis of FRN during simple versus complex trials. Unfortunately, the amount of trials and subsequent free-artifact EEG segments impede this goal. Consequently, this limitation should be addressed in future studies.

Regarding fP300, there is a clear effect of agent (higher voltages for subject’s own performance compared to peers) that is similar to the effect reported by Leng and Zhou [33], quite probably due to an increased allocation of attentional resources on the participant’s own performance. The relation of P300 to attentional processes has been widely documented in literature [32]. Also, an interaction with valences seems to arise: although voltages are higher when a subject is reviewing her/his own performance, the effect is particularly strong when feedback indicates a correct response.

FRN and fP300 have the same scalp distribution that has been reported in previous experiments; *i*.*e*., marked at midline, but more frontal for FRN and more parietal for fP300. It is very likely that the same sources of neural activity that have been reported to underlie these components do the same in our task. In that case, our results would be in line with the reward theory, which states that FRN somehow reflects detection that performance was “worse than expected”. One especially interesting result is that FRN would be elicited regardless of its performance regulation value: in our task, the ACC activity that underlies FRN does not imply regulation, since the observed performance is no longer modifiable.

Based on the present results, two main points should be emphasized: a) the nature of the task, in which subjects–based on the serial precedent of changing variables–could predict the closing stimulus of each trial by constructing a mental template that might match, or not, the incoming stimulus; and, b) the design of an experimental paradigm that allows the recording of feedback-related electrophysiological responses from non-contingent feedback that is attributed to earlier performance on a task. The first of these characteristics allowed us to use a large number of distinct trials that make the future review of performance interesting enough to obtain the required feedback-related ERP components. The second opens up new possibilities of experimentation for vicarious monitoring in which there is no need for performer and observer to be present during the task, but still allows measurement of the reaction to errors attributed to peers.

Obtaining feedback-related components from non-active performance has been reported before: e.g. Yeung, Holroyd and Cohen [25] showed that the component could be obtained using a gambling task in which the subject made no choices but only received feedback on gains and losses. One technical drawback of this kind of task is the need to maintain the participant’s attention while she/he is not taking active part in the task. To control for this, we included blocks with a simple-choice gambling task, but in future research it might be advisable to explore tasks in which trial duration is shorter, so that the experiments can include more trials without detaining subjects for long periods of time. Also, as we are obtaining ERPs after the participants review trials of a task they previously performed, they might be covertly attempting to “respond” to the task by creating a mental image of the expected response, thereby overlapping response and feedback related activity on the EEG. Further developments would have to consider using only complex trials, in order to address the possibility of feedback reflecting not only information about performance but about *expected* performance. An additional line of research could use a similar model (*i*.*e*., participants receive non-contingent feedback related to the performance of distinct agents) but with different tasks. In this case it would be important to assure that the trials differ so as to maintain the subject’s attention and the information-value of feedback.
